# Osteochondral Alterations in Patients Treated with Total Knee Arthroplasty Due to Rheumatoid Arthritis and Primary Osteoarthritis: Cross-Sectional Study with Focus on Elucidating Effects of Knee Malalignment

**DOI:** 10.3390/life15050818

**Published:** 2025-05-20

**Authors:** Andreja Baljozovic, Aleksa Lekovic, Slobodan Nikolic, Danijela Djonic, Marija Djuric, Zoran Bascarevic, Jelena Jadzic

**Affiliations:** 1 Institute for Orthopaedics Banjica, Faculty of Medicine, University of Belgrade, Mihaila Avramovica 28, 11000 Belgrade, Serbia; a.baljozovic@gmail.com (A.B.); zoran.bascarevic@iohbb.edu.rs (Z.B.); 2 Institute of Forensic Medicine, Faculty of Medicine, University of Belgrade, Deligradska 31a, 11000 Belgrade, Serbia; aleksa.lekovic@med.bg.ac.rs (A.L.); slobodan.nikolic@med.bg.ac.rs (S.N.); 3 Center of Bone Biology, Faculty of Medicine, University of Belgrade, Dr. Subotica 4/2, 11000 Belgrade, Serbia; danijela.djonic@med.bg.ac.rs (D.D.); marijadjuric5@gmail.com (M.D.)

**Keywords:** rheumatoid arthritis, knee osteoarthritis, tibia, femur, micro-CT, subchondral bone, cartilage, total knee arthroplasty, knee alignment

## Abstract

Micro-computed tomography assessment of osteochondral microstructural properties of the distal femur and proximal tibia was comprehensively conducted to compare adult patients with knee rheumatoid arthritis (RA) and primary knee osteoarthritis (KOA), with special focus on the effects of knee malalignment. This study encompassed 402 bone samples divided into three groups: the RA group [patients who were subjected to total knee arthroplasty (TKA) due to RA, *n* = 23, age: 61 ± 10 years], the KOA group [individuals subjected to TKA due to KOA, *n* = 24, age: 71 ± 9 years] and the control group [sex-matched cadavers without degenerative knee diseases, *n* = 20, age: 67 ± 11 years]. Our data revealed that the RA, KOA, and control groups differ significantly in osteochondral microstructural properties depending on the knee alignment. Specifically, increasing femoral and tibial cortical porosity, coupled with thinner articular cartilage, were noted in the RA and KOA groups, compared to the controls. Furthermore, larger femoral and tibial cortical pores, lower tibial and femoral subchondral trabecular bone fraction, and thinner tibial articular cartilage were noted in the RA group in comparison to the KOA group, implying that the medial-to-lateral load distribution in the knee joint could be most affected in these patients. Our data illustrated that the thinnest cartilage, a thicker and less porous cortex, along with lower trabecular bone volume, were present in the lateral femoral and tibial condyles of RA individuals with valgus knee alignment. Observed subchondral trabecular microarchitectural alterations could be morphological factors contributing to different effects of surgical treatment and variable implant stability in individuals with RA, warranting further research.

## 1. Introduction

Knee osteoarthritis (KOA) is a chronic progressive condition that represents a major global public health concern as one of the leading causes of disability that frequently requires surgical treatment [1]. It can be classified as primary KOA, meaning it is an idiopathic degenerative condition primarily occurring with aging, or secondary KOA, where knee joint cartilage damage is caused by previous injuries, infections, or inflammatory arthropathies, such as rheumatoid arthritis (RA) [2].

Initially, it was thought that KOA primarily affects articular cartilage, while it is now understood as a disease that affects all structures, disabling the physiological functioning of the synovial joint. Recent research suggests that subchondral bone (SB) plays a significant role in KOA etiopathogenesis [3,4]. It consists of two anatomically distinct structures: the subchondral cortical bone plate (SBP, representing a thin cortical bone lamella enabling communication between articular cartilage and underlying bone) and the subchondral trabecular bone (STB, consisting of supporting trabeculae beneath SBP that play a significant role in load-bearing and stress-absorption) [5,6]. This tissue compartment exhibits high metabolic activity and plays a crucial role in supplying nutrients to the overlaying articular cartilage [7]. Together with articular cartilage, they form a specialized unit called the osteochondral junction, where biomechanical and biochemical interactions occur [8]. Alterations occurring in a single osteochondral junction component cannot be evaluated separately, as they influence the entirety of the assembly, indicating that comprehensive osteochondral evaluation is of paramount importance to comprehend arthritic alterations in the knee joint [6]. Further, it has been noted that knee alignment significantly affects load distribution across the femorotibial compartments. When misaligned, the load axis shifts eccentrically, creating a moment arm that increases the load on one compartment, which is a critical factor in the KOA development [9].

Although the contemporary literature contains a substantial amount of research about the hip microstructural osteochondral characteristics [10,11], quantitative characterization of the regional distribution of the SB microarchitecture of the proximal tibia and distal femur in RA and KOA remains insufficiently explored [11,12]. The available data suggest that in both joints, the bone density increases in regions closely associated with damaged cartilage [1]. Previous studies have shown that degenerately altered cartilage is less capable of absorbing forces than healthy cartilage. As the condition advances, a harmful cycle begins, characterized by the extent of deformity and subchondral sclerosis that occurs in response to this loading, which, in turn, exacerbates the progression of the deformity [13,14]. Still, there is no agreement on whether the modified SB microarchitecture initiates disease onset or develops as a secondary effect of cartilage degeneration [4]. Further, managing advanced stages of the disease typically requires total knee arthroplasty (TKA), which is generally effective, but ongoing efforts are necessary to enhance the longevity of the implants, particularly in terms of their bone fixation stability. Comprehensive evaluation of microstructural knee joint properties is crucial to ensuring that this fixation remains durable across various patient populations [14]. Previous studies on microstructural alterations in RA/KOA patients [14,15,16,17,18,19,20,21,22] are limited by various factors: different diagnostic criteria for RA/KOA, the variable number of participants, including only one group of patients, or including exclusively individuals with RA or KOA when compared to controls, neglecting the importance of covariant factors (sex, age, body size, physical activity, etc.), analyzing just one skeletal site (femur or tibia), and using variable methodologies limiting inter-study comparability (low scanning resolution, two dimensional assessment, or limited assessed bone volume), which indicates the need for more detailed research.

Thus, the primary aim of this study was to conduct a comprehensive high-resolution comparative assessment of SB microstructural and cartilage properties of the distal femur and proximal tibia between adult patients with RA treated with TKA, patients with KOA treated with TKA, and control individuals without degenerative knee diseases. A secondary aim of this study was to assess the potential site-specificity in RA/KOA-induced changes in SB microstructural and cartilage properties of the distal femur and proximal tibia, depending on knee malalignment.

## 2. Materials and Methods

### 2.1. Study Design and Patient Selection Process

**Figure 1:**
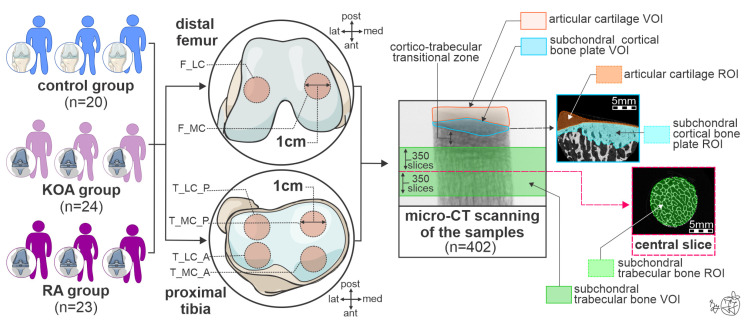
Schematic representation of the methodological steps used in this study to assess subchondral bone microstructure and articular cartilage. For the interpretation of the abbreviations used in the figure, readers are referred to the list of abbreviations. The figure represents the author’s original artwork, hand-generated using vector graphic editor software (CorelDRAW Graphics Suite 24.3 version, Cascade Parent Limited, Ottawa, ON, Canada) and signed by the creator.

This cross-sectional study comprised a total of 402 bone samples (268 proximal tibia and 134 distal femur samples) collected from 67 adult individuals divided into three groups: the control group, the KOA group, and the RA group (Figure 1).

The KOA group consisted of individuals requiring TKA due to KOA who were treated at the Institute for Orthopaedics Banjica between 2023 and 2025 (*n* = 24). The RA group consisted of individuals requiring TKA due to knee RA who were treated at the Institute for Orthopaedics Banjica between 2023 and 2025 (*n* = 23). The initial RA and KOA diagnoses were based on the patient’s medical history and clinical presentation in reference to findings on preoperative anteroposterior and lateral knee radiographs. All patients in the RA group were previously diagnosed by a rheumatologist as seropositive RA and treated with conventional and biological disease-modifying antirheumatic drugs (DMARDs) in combination with corticosteroids in some patients (Table 1). Preoperative knee radiographs were conducted to confirm radiographic features of the RA/KOA (knee joint space narrowing, osteophyte formation, to assess presence of subchondral sclerosis, presence of cystic formations, and bone deformity), to provide a Kellgren–Lawrence grade (illustrating the severity of RA/OA lesions) and to measure the hip–knee–ankle (HKA) angle. A positive angle value indicated valgus, while a negative value indicated varus alignment. To minimize inter-observer variability, a single investigator interpreted knee radiographs and conducted HKA measurements in the Imaging Clinical Information System (ICIS, Agfa HealthCare, Mortsel, Belgium). Control group samples were collected from sex-matched cadaveric donors without macroscopic appearance and heteroanamnestic/medical data about degenerative knee diseases (*n* = 20). Based on a detailed review of autopsy reports, the leading causes of death of these individuals were cardiovascular diseases (including acute cardiac failure, stroke, and cerebral hemorrhage) and traffic accidents. All individuals included in the study were from similar socio-economic contexts and cultural backgrounds.

Individuals with a positive history of bone-affecting diseases (chronic liver or renal disease, dysfunction of the parathyroid, suprarenal glands, and gonads, the presence of solitary and metastatic cancerous lesions, the presence of osteomyelitis and other bone-affecting infections, data about permanent immobility and being bed-ridden, and data about previous fragility fracture), as well as the use of anti-resorptive drugs, antiepileptics, chemotherapy, or hormonal replacement therapy were not eligible for this study. Further, we excluded patients with traumatic knee joint injury, patients with ligament instability, patients with physical signs of malnourishment (body mass index [BMI] below 18.5 kg/m^2^), and patients who were unable to self-consent or who had a diagnosis of any cognitive impairment. Patients who underwent intraarticular knee treatments (viscosupplementation or corticosteroid injections) and individuals prone to chronic alcoholism or substance abuse were not eligible for this study. Human remains in an advanced stage of decomposition were not considered eligible for this study. The basic data for the included individuals are presented in Table 1.

### 2.2. Bone Sampling Process and Storage

Using an osteochondral autograft transfer system harvester (OATS, Arthrex, Naples, FL, USA), 1 cm-thick cylindrical bone samples were obtained from the distal femur and proximal tibia. Based on the rigorous following of the intraoperative TKA technique and previous reports that the central and thickest region of the resected condyles exerts the greatest loads [23], we planned a bone sample collection (Figure 1). Namely, a total of six bone samples per person were harvested (marked with brick red circles in Figure 1): two samples of distal femora (one from the medial femoral condyle [F_MC] and one from the lateral femoral condyle [F_LC]), and four samples of proximal tibia (one from the anterior [T_MC_A], one from the posterior part of the medial femoral condyle [T_MC_P], one from the anterior [T_LC_A], and one from the posterior part of lateral tibial condyle [T_LC_P]). To ensure adequate tissue fixation, bone samples were stored in a standard formaldehyde solution (i.e., 10% neutral buffered formalin) for at least two weeks.

### 2.3. High-Resolution 3D Histomorphometric Assessment of Knee Osteochondral Properties

This study followed our previous recommendations to ensure adequate imaging quality and reliable 3D histomorphometric assessment standards [24]. In short, each bone sample was consistently placed on the sample holder, foam-fixed to prevent movement of the artifacts, wrapped in parafilm to prevent tissue desiccation, and scanned using the Skyscan 1172 micro-computed tomography system (Bruker micro-CT, Skyscan, Kontich, Belgium). The system operated under the following scanning protocol: spatial resolution of 10 µm, 80 kV, 126 μA, Al + Cu filter, 2 K camera binning, 1200 ms exposure time, 0.4° rotation step, and triple frame averaging. Afterwards, projection images were reconstructed using NRecon software (1.7.4.6 version, Skyscan, Kontich, Belgium) with appropriate corrections for misalignment, thermal drift, ring artifacts, and beam hardening. Then, reconstructions were saved as 8-bit bitmap format images (256 gray levels, gray levels between 95 and 255 were considered as bone tissue). The correctness of the applied thresholds was inspected by comparing them with the original images. The same threshold value was used for all the samples to allow inter-individual comparisons.

As presented in Figure 1, the segmentation procedure involved manually contouring the regions of interest (ROIs) on various slices, with interpolation applied to produce 3-dimensional volumes of interest (VOIs). VOIs of SBP comprised 300 manually demarcated ROIs (approximately 0.3 cm^3^ per sample, marked with a blue square in Figure 1). Based on previous reports [21] that the most prominent STB alterations are predominantly located 5 mm away from the outer cortical shell (central slice, marked with a pink dashed line in Figure 1), a total of 701 slices (central slice ± 350 slices, approximately 0.7 cm^3^ per sample) were included in each STB VOI (marked with a green square in Figure 1). To minimize inter-observer variability, a single investigator experienced in micro-CT assessment conducted VOI demarcation procedures.

The following microarchitectural SBP parameters were analyzed using the newest 64-bit CT-Analyzer software (CT.An 2020; 1.20.30.0 version, Skyscan, Kontich, Belgium): total cortical porosity (Ct.Po.tot, %), cortical pore separation (Ct.Po.Sp, mm), cortical pore diameter (Ct.Po.Dm, µm), and cortical thickness (Ct.Th, mm). Moreover, the following STB microarchitectural parameters were analyzed: trabecular bone volume fraction (BV/TV, %), trabecular number (Tb.N, 1/mm), trabecular thickness (Tb.Th, µm), trabecular separation (Tb.Sp, mm), connectivity density (Conn.Dn, 1/mm^3^), fractal dimension (FD, dimensionless), degree of anisotropy (DA, dimensionless), and structure model index (SMI, dimensionless). Standard guidelines for histomorphometric nomenclature developed by the American Society for Bone and Mineral Research (ASBMR) were applied in the present study [25].

Articular cartilage was segmented on the same 8-bit bitmap format reconstruction images of the samples, using a threshold window of 20 and 95 to exclude air, bone marrow, and bone tissue. The cartilage segmentation was verified manually (marked with an orange square in Figure 1), and a “shrink-wrap” plug-in was applied to confirm the boundaries of the cartilage ROIs [20]. Using the newest 64-bit CT-Analyzer software (CT.An 2020; 1.20.30.0 version, Skyscan, Kontich, Belgium), we analyzed cartilage thickness (Cg.Th, µm) in harvested bone samples.

### 2.4. Statistical Analysis

The Kolmogorov–Smirnov test was used to assess the normality of the data distribution, while Levine’s test was employed to verify the homogeneity of the data variance. To omit the covariant effects of age and BMI on our results [26,27], analysis of covariance (ANCOVA) with Bonferroni post hoc correction for pairwise comparisons was used to evaluate potential differences between the investigated groups (RA, KOA, and control group), between different knee malalignment subgroups (varus and valgus knee alignment), between investigated skeletal sites (F_LC, F_MC, T_MC_A, T_MC_P, T_LC_A, and T_LC_P), and their interaction (the skeletal site was set as a within-subject factor, and the group was set as a between-subject factor). The medial-to-lateral ratios of osteochondral microstructural parameters (BV/TV for STB, Ct.Th for SBP, and Cg.Th for cartilage) were calculated to provide insights into load distribution within the knee joint [4]. Statistical analysis was performed in statistical software (IBM SPSS Statistics, 27.0 version, International Business Machines Corporation, Armonk, NY, USA) at a 0.05 significance level and 95% confidence interval (*p* < 0.05).

## 3. Results

### 3.1. Comparisons of Femoral Osteochondral Microstructure in Individuals with RA, Individuals with KOA, and Controls

**Figure 2:**
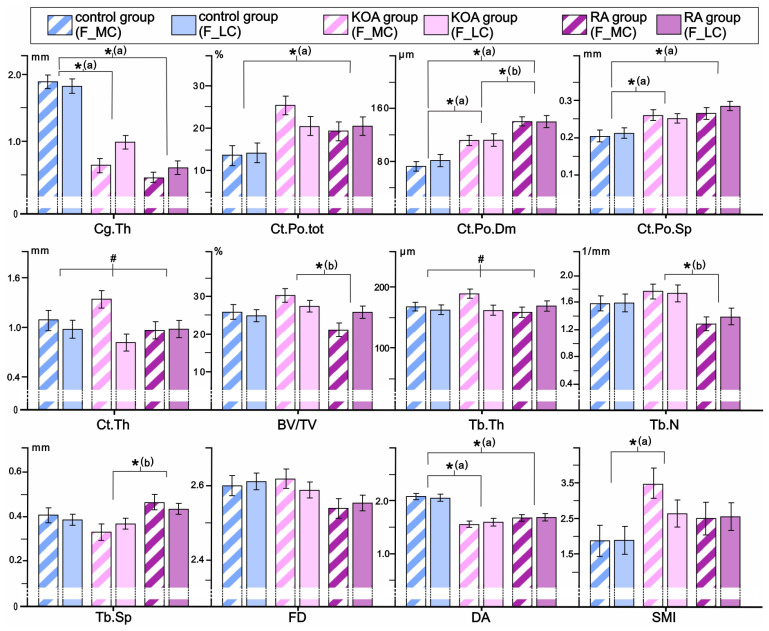
Comparison of osteochondral microstructural parameters in the distal femora of individuals with RA, individuals with KOA, and controls. The statistical significance of the inter-group differences was estimated using ANCOVA (covariates in the model are evaluated at the following values: age: 66.82 years and BMI: 27.84 kg/m^2^) with Bonferroni post hoc correction for pairwise comparisons (* group-*p* < 0.05 [a vs. control, b vs. KOA group], # site × group-*p* < 0.05). Bar graphs represent the data as mean ± SE. For the interpretation of the abbreviations used in the figure, readers are referred to the list of abbreviations.

Our data revealed that the RA, KOA, and control groups differ significantly in femoral SB microarchitectural and cartilage properties even after adjustment for BMI and age (Figure 2). Namely, significant inter-group differences were noted for Ct.Po.tot (group-*p* = 0.005), Ct.Po.Dm (group-*p* = 0.001), Ct.Po.Sp (group-*p* = 0.001), Cg.Th (group-*p* = 0.001), BV/TV (group-*p* = 0.019), Tb.N (group-*p* = 0.035), Tb.Sp (group-*p* = 0.020), DA (group-*p* = 0.001), and SMI (group-*p* = 0.046) (Figure 2). Bonferroni post hoc correction revealed a substantial increase in Ct.Po.tot (*p* = 0.004), Ct.Po.Dm (*p* = 0.001), and Ct.Po.Sp (*p* = 0.001), coupled with lower Cg.Th (*p* = 0.001) and DA (*p* = 0.001) in femoral samples of the RA group when compared to controls. Further, Bonferroni post hoc correction revealed a substantial increase in femoral Ct.Po.Dm (*p* = 0.02), Ct.Po.Sp (*p* = 0.016), and SMI (*p* = 0.042), coupled with lower DA (*p* = 0.001) and Cg.Th (*p* = 0.001) in individuals with KOA compared to controls. As shown in Figure 2, femoral samples of the RA group differed significantly from the KOA group by high Ct.Po.Dm (*p* = 0.017) and Tb.Sp (*p* = 0.016), along with lower BV/TV (*p* = 0.017) and Tb.N (*p* = 0.032). The site × group interaction was significant only for Ct.Th (site × group-*p* = 0.013) and Tb.Th (site × group-*p* = 0.020), while the tendency to significant interaction was noted for BV/TV (site × group-*p* = 0.067), illustrating the most prominent negative effect on femoral bone microstructure in individuals with RA (Figure 2).

### 3.2. Inter-Site Differences in Femoral Osteochondral Parameters as Indicators of Medial-to-Lateral Load Distribution in the Knee Joint of Individuals with RA and KOA Depending on Knee Alignment

As shown in Table 2, a more detailed analysis revealed site-specific alterations in femoral SB microarchitectural and cartilage properties depending on knee malalignment in individuals with RA and KOA. Namely, the site × subgroup interaction was significant for Cg.Th, Ct.Po.tot, Ct.Th, BV/TV, and Tb.Th (site × subgroup-*p* < 0.05, Table 2), illustrating the thinnest cartilage, a thicker and less porous cortex, along with lower BV/TV and Tb.Th in the lateral femoral condyle of individuals with RA and valgus knee alignment (Table 2). These data are supported by the highest medial-to-lateral ratio of BV/TV recorded in individuals with RA and valgus knee alignment (Table 2). Further, these significant interactions illustrated the highest BV/TV values and the thickest cortex in the medial femoral condyle of individuals with KOA and varus knee alignment (Table 2), which was supported by an inclining trend in BV/TV and Ct.Th medial-to-lateral ratios of these individuals. Knee alignments did not significantly affect remaining microarchitectural parameters (site × subgroup-*p* > 0.05 and subgroup-*p* > 0.05).

### 3.3. Comparisons of Tibial Osteochondral Microstructure in Individuals with RA, Individuals with KOA, and Controls

**Figure 3:**
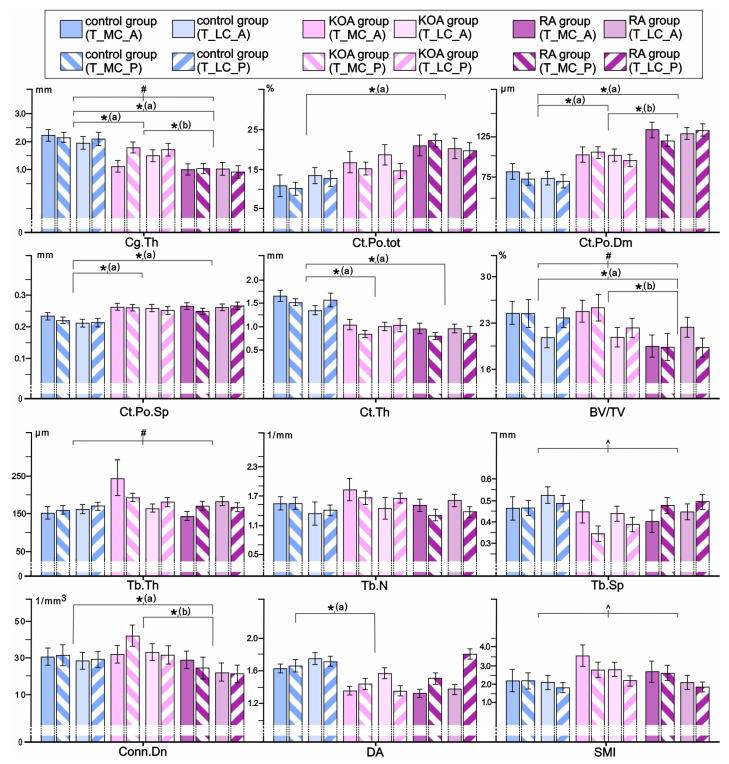
Comparison of osteochondral microstructural parameters in the proximal tibia of individuals with RA, individuals with KOA, and controls. The statistical significance of the inter-group differences was estimated using ANCOVA (covariates in the model are evaluated at the following values: age: 66.82 years and BMI: 27.84 kg/m^2^) with Bonferroni post hoc correction for pairwise comparisons (* group-*p* < 0.05 [a vs. control, b vs. KOA group], ^ site-*p* < 0.05, # site × group-*p* < 0.05). Bar graphs represent the data as mean ± SE. For the interpretation of the abbreviations used in the figure, readers are referred to the list of abbreviations.

Our data revealed that RA, KOA, and control groups differ significantly in tibial SB microarchitectural and cartilage properties even after adjustment for age and BMI (Figure 3). Namely, significant inter-group differences were noted for Ct.Po.tot (group-*p* = 0.002), Ct.Po.Dm (group-*p* = 0.001), Ct.Po.Sp (group-*p* = 0.001), Ct.Th (group-*p* = 0.001), Cg.Th (group-*p* = 0.001), BV/TV (group-*p* = 0.012), Conn.Dn (group-*p* = 0.032), and DA (group-*p* = 0.001). Bonferroni post hoc correction revealed a substantial increase in Ct.Po.tot (*p* = 0.002), Ct.Po.Dm (*p* = 0.001), and Ct.Po.Sp (*p* = 0.001), coupled with lower Cg.Th (*p* = 0.001), Ct.Th (*p* = 0.001), BV/TV (*p* = 0.006), and Conn.Dn (*p* = 0.030) in the tibia of the RA group when compared to controls. Further, Bonferroni post hoc correction revealed a substantial increase in tibial Ct.Po.Dm (*p* = 0.03), Ct.Po.Sp (*p* = 0.002), coupled with lower Cg.Th (*p* = 0.001), Ct.Th (*p* = 0.001), and DA (*p* = 0.001) in individuals with KOA compared to controls. As shown in Figure 3, tibial samples of the RA group differed significantly from the KOA group by high Ct.Po.Dm (*p* = 0.02), along with lower Cg.Th (*p* = 0.008), BV/TV (*p* = 0.046), and Conn.Dn (*p* = 0.026). The site × group interaction was significant only for Cg.Th (site × group-*p* = 0.047), BV/TV (site × group-*p* = 0.041), and Tb.Th (site × group-*p* = 0.043), illustrating the most prominent negative effect on tibial bone microstructure in individuals with RA (Figure 3).

### 3.4. Inter-Site Differences in Tibial Osteochondral Microstructural Parameters as Indicators of Medial-to-Lateral Load Distribution in the Knee Joint of Individuals with RA and KOA Depending on Knee Alignment

As shown in Table 3, a more detailed analysis revealed site-specific alterations in tibial SB microarchitectural and cartilage properties, depending on knee malalignment, in individuals with RA and KOA who underwent TKA.

Namely, site × subgroup interaction was significant for Cg.Th, Ct.Th, and BV/TV (site × subgroup-*p* < 0.05, Table 3), illustrating that lateral tibial compartments of individuals with RA and valgus knee alignment had thin cartilage and thicker cortex, along with lower BV/TV (Table 3). These data are supported by the highest medial-to-lateral ratio of Cg.Th and the lowest medial-to-lateral ratio of Ct.Th recorded in individuals with RA and valgus alignment (Table 3). Further, these significant interactions illustrated that medial tibial compartments of individuals with KOA and varus knee alignment had the highest BV/TV values and the thickest cortex (Table 3). Other assessed microarchitectural parameters were not significantly affected by knee alignments (site × subgroup-*p* > 0.05 and subgroup-*p* > 0.05).

## 4. Discussion

In contrast to individuals with primary KOA, the contemporary literature lacks micro-scale osteochondral assessment in individuals with knee RA. To the best of our knowledge, this is the first study on knee SB and cartilage microstructural changes conducted in patients with KOA, RA, and controls, using comprehensive high-resolution micro-CT analysis. In contrast to previous studies, we comprehensively analyzed femoral and tibial samples to investigate RA/KOA-induced micro-scale osteochondral changes in relation to knee malalignment.

Our data indicated that femoral and tibial cartilage were significantly thinner in individuals with RA and KOA compared to controls, with tibial cartilage being thinner in the RA group compared to the KOA group, which is consistent with previous findings [14]. Our data suggested that femoral cartilage thinning in the RA group was more pronounced compared to cartilage covering the tibial plateau. These data are in agreement with the previous MRI study involving 26 patients with RA, which reported that cartilage damage is one of the characteristic lesions most pronounced on the lateral femoral condyle [22]. Further, our data also revealed significant differences in SBP and STB microstructure in individuals with RA and KOA compared to controls. Femoral and tibial samples of the RA and KOA groups showed increasing cortical porosity coupled with lower tibial cortical thickness compared to the control group. Furthermore, larger femoral and tibial cortical pores coupled with lower STB fraction and fewer trabeculae were noted in RA patients compared to the KOA group, implying that a negative effect on the tibial and femoral SB morphology was mostly pronounced in patients with RA. The study conducted by Song et al. was the only previous study comparing knee SB microstructure between individuals with KOA and RA [28]. This study analyzed only the femoral SB microstructure, revealing impaired bone structure in the RA group compared to KOA, in accordance with our results. These findings could be explained by the effects of RA-induced inflammatory infiltrate and pro-inflammatory cytokines accumulation, which contribute to increased osteoclast activity and bone destruction due to increased bone resorption [10]. It is worth noting that we did not observe any significant differences between the anterior and posterior samples of the tibial plateau in individuals with RA/KOA. This can be explained by the exclusion of patients with ligamentous instability. Ligamentous injuries lead to uneven loading, which is especially evident for anterior cruciate ligament tears and the consequent anterior translation of the tibia during walking, which results in a direct increase in pressure on the posterior aspects of the knee joint surfaces, especially in the posterolateral compartment [29]. On the other hand, Song et al. also implied that cement penetration may be better in RA patients due to their more porous bone structure [28], while others revealed that impaired microstructure has an adverse effect on implant stability [30]. Huang et al. reported that patients with lower trabecular bone volume fraction, higher Tb.Sp, and higher SMI experienced less pain in the early postoperative period of TKA [30]. However, the positive contribution of deteriorated trabecular microarchitecture to prosthesis fixation would significantly deteriorate with time [30]. Since SBP and cartilage would be surgically removed during TKA, implant fixation is achieved at the level of STB, whose microstructure is responsible for prosthesis stability and longevity. Our data on a lower STB fraction and fewer trabeculae in the region of surgical resection, predominantly noted in RA patients, suggest that these morphological factors may affect the fixation stability of surgical implants over time. However, recent meta-analyses have revealed conflicting data regarding the revision rate and risk of prosthetic loosening in patients with RA compared to KOA [31,32], indicating that further research is warranted to elucidate the clinical relevance of these microstructural findings.

Importantly, our study revealed significant femoral and tibial osteochondral variations in patients with RA/KOA in relation to knee malalignment. Namely, our data indicated an RA/KOA-induced inverse relationship of Cg.Th and BV/TV, and a direct relationship of the tibial Ct.Th in the lateral tibial condyles of individuals with valgus alignment and in the medial tibial condyles of individuals with varus alignment, which was further supported by the differences in the medial-to-lateral ratios. Interestingly, only the BV/TV medial-to-lateral ratio was significantly different in femoral samples, whereas in the tibia, this difference was observed for BV/TV, Cg.Th, and Ct.Th medial-to-lateral ratios. Therefore, these results may suggest that the tibial plateau has a more heterogeneous microarchitecture, which is more affected by knee malalignment compared to the femoral condyles. Han et al. showed that abnormal local stress distribution between the medial and lateral knee compartments results in cartilage mechanical damage and abnormal trabecular bone remodeling, and that knee malalignment is a significant risk factor for the occurrence and development of KOA [9,33]. Robins et al. pointed to a clear connection between knee loading and reduction in Cg.Th in the KOA group compared to healthy controls [34]. Furthermore, Takeda et al. reported that frequently observed valgus alignment represents one of the characteristics in the clinical presentation of knee RA [35], which is in accordance with our observations. Our data suggests that knee malalignment is associated with higher Ct.Th in the medial compartments of individuals with varus deformity and higher Ct.Th in the lateral compartments in individuals with valgus deformity. This finding confirms that during the pathogenesis of degenerative knee disease, there is a disruption of the harmonious balance of joint load influenced by the alignment and SBP morphology [14]. Nevertheless, our results regarding STB bone mass differ from previous studies that reported higher BV/TV on the loaded side of the joint [14,17,20]. These inconsistencies could be, at least partially, explained by the covariant effect of the transitional cortico-trabecular zone (Figure 1) that was included in the microstructural analysis in previous studies. However, current recommendations for human bone microstructural analysis advocate for excluding the transitional cortico-trabecular zone to ensure better inter-study comparability [24]. One of the strengths of our study is the analysis of the STB closest to the resection lines during TKA, which most realistically describes the microstructure of the actual implant fixation bone surface. Thus, it is indicative that further research is warranted to fully understand the effects of knee malalignment on STB microstructural alterations and its role in implant stability in patients with RA and KOA.

This study has some specific limitations that should be acknowledged. We designed a cross-sectional observational study, meaning that assessing temporal changes in osteochondral knee features was not possible over time. We conducted detailed analyses to exclude covariate effects of various factors (i.e., age, BMI, sex), but we could not fully address the potential confounding effect of unidentified and unreported habits (e.g., different physical activity levels, smoking status, poor nutrition, vitamin D deficiency). To adequately illustrate real-life situations in the clinical management of knee RA/KOA, the potential covariate effect of antirheumatic therapy in investigated individuals could not be avoided (Table 1). Our study included only individuals requiring surgical treatment due to advanced stages of KOA/RA (Table 1), indicating that our results may not be fully generalizable to the population of patients with early-stage disease. Our micro-CT assessment of knee joint cartilage did not involve the use of contrast agents, allowing for morphological assessment but not compositional cartilage evaluation. Thus, future studies would benefit from using contrast agents to provide data about RA/KOA-induced alterations in cartilage proteoglycan content [36]. Furthermore, we exclusively focused on using micro-CT to assess SB microarchitectural parameters as a marker of bone strength, thereby indirectly estimating skeletal status in RA/KOA patients. Thus, future studies would benefit from employing a multi-scale approach in assessing other hierarchical bone organization features, combining experimental and simulation studies to elucidate the puzzling mechanisms underlying RA/KOA-induced SB alterations [37]. Considering that our study design involves cadaveric donors in whom bone turnover biomarkers serum concentrations are unreliable, we could not estimate the relative contributions of increased bone resorption and decreased bone formation in the skeletal disturbances observed in our individuals. Thus, future studies focusing on bone histology would benefit from using dynamic histomorphometry with tetracycline labeling to assess the precise RA/KOA-induced changes in bone remodeling.

## 5. Conclusions

Our data revealed that the RA, KOA, and control groups differ significantly in osteochondral properties depending on the knee malalignment. The inverse relationship between cartilage thickness and SB properties revealed morphological traces of the knee joint response to mechanical load, implying that these microstructural differences may be helpful indicators of disease progression in patients with RA/KOA, warranting further research. Further, we noted site-specific microstructural alterations in patients with RA compared to other groups, implying that the medial-to-lateral load distribution in the knee joint could be most affected in these patients. Site-specificity of SB microarchitectural alterations could be morphological factors contributing to different effects of surgical treatment and variable implant stability in individuals with knee RA, warranting further research.

## Figures and Tables

**Table 1:** Basic clinical data of individuals included in our study.

	Control Group (*n* = 20)	KOA Group (*n* = 24)	RA Group (*n* = 23)
Basic anthropometric characteristics			
Age (years)	67 ± 11	71 ± 9	61 ± 10
Height (cm)	172 ± 10	169 ± 10	163 ± 8
Weight (kg)	75 ± 22	85 ± 14	74 ± 13
BMI (kg/m^2^)	25.1 ± 6.8	29.8 ± 4.3	28.1 ± 5.1
Sex	12/20 F 8/20 M	17/24 F 7/24 M	19/23 F 4/23 M
Radiographic characteristics			
Kellgren–Lawrence classification	N.A.	0/24 grade 1 1/24 grade 2 10/24 grade 3 13/24 grade 4	0/23 grade 1 1/23 grade 2 8/23 grade 3 14/23 grade 4
Varus knee alignment	N.A.	14/24	8/23
Valgus knee alignment	N.A.	10/24	15/23
HKA angle (°)	N.A.	−2.27 ± 9.19	4.42 ± 12.45
Chronic comorbidities			
Hypertension	16/20	23/24	13/23
CAD	13/20	4/24	2/23
PVD	4/20	4/24	1/23
HLP	N.A.	3/24	3/23
T2DM	3/20	7/24	5/23
Thyroid disorders	0/20	5/24 HT	3/23 HT
Antirheumatic pharmacotherapy			
Glucocorticoids	N.A.	N.A.	9/23
Methotrexate and other DMARDs	N.A.	N.A.	20/23
Biological treatment	N.A.	N.A.	6/23

Quantitative parametric data are expressed as mean ± SD, while ordinal and nominal data are reported as a ratio of the total (*n*/max). For the interpretation of abbreviations used in the table, readers are referred to the list of abbreviations.

**Table 2:** Femoral cartilage and SB microstructural parameters in individuals with RA and KOA depending on knee malalignment.

Skeletal Site		KOA Group Valgus Knee (Mean ± SE)	KOA Group Varus Knee (Mean ± SE)	RA Group Valgus Knee (Mean ± SE)	RA Group Varus Knee (Mean ± SE)	*p*-Value
Cg.Th (mm)	F_MC	0.67 ± 0.12	0.46 ± 0.11	0.55 ± 0.11	0.44 ± 0.13	subgroup-*p* = 0.169 site-*p* = 0.471 **site × subgroup-*p* = 0.003**
	F_LC	0.93 ± 0.14	1.08 ± 0.12	0.39 ± 0.13	0.96 ± 0.15	
Medial-to-lateral ratio of Cg.Th		0.72 ± 0.23	0.43 ± 0.22	1.41 ± 0.19	0.46 ± 0.26	subgroup-*p* = 0.107
Ct.Po.tot (%)	F_MC	19.73 ± 3.48	19.86 ± 2.99	22.58 ± 3.18	28.72 ± 3.73	subgroup-*p* = 0.378 site-*p* = 0.079 **site × subgroup-*p* = 0.001**
	F_LC	28.74 ± 3.10	16.58 ± 2.66	19.24 ± 2.83	17.42 ± 3.32	
Ct.Po.Dm (µm)	F_MC	113 ± 12	113 ± 10	134 ± 11	137 ± 12	subgroup-*p* = 0.200 site-*p* = 0.440 site × subgroup-*p* = 0.175
	F_LC	131 ± 14	102 ± 12	142 ± 13	118 ± 15	
Ct.Th (mm)	F_MC	0.96 ± 0.19	1.50 ± 0.15	0.92 ± 0.16	1.29 ± 0.18	subgroup-*p* = 0.416 site-*p* = 0.101 **site × subgroup-*p* = 0.050**
	F_LC	0.92 ± 0.18	0.91 ± 0.14	1.06 ± 0.15	0.70 ± 0.16	
Medial-to-lateral ratio of Ct.Th		1.29 ± 0.31	2.04 ± 0.29	0.88 ± 0.26	1.52 ± 0.36	subgroup-*p* = 0.101
Tb.Th (µm)	F_MC	160 ± 13	179 ± 11	192 ± 11	167 ± 14	subgroup-*p* = 0.817 site-*p* = 0.663 **site × subgroup-*p* = 0.015**
	F_LC	174 ± 12	151 ± 10	163 ± 12	181 ± 13	
Tb.N (1/mm)	F_MC	1.625 ± 0.142	1.993 ± 0.122	1.519 ± 0.177	1.353 ± 0.152	**subgroup-*p* = 0.014** site-*p* = 0.317 site × subgroup-*p* = 0.351
	F_LC	1.585 ± 0.194	1.895 ± 0.207	1.248 ± 0.130	1.292 ± 0.207	
Tb.Sp (mm)	F_MC	0.330 ± 0.053	0.292 ± 0.045	0.526 ± 0.048	0.378 ± 0.057	**subgroup-*p* = 0.033** site-*p* = 0.494 site × subgroup-*p* = 0.189
	F_LC	0.348 ± 0.043	0.371 ± 0.037	0.440 ± 0.039	0.425 ± 0.046	
BV/TV (%)	F_MC	27.57 ± 2.96	34.42 ± 2.54	27.38 ± 2.40	23.24 ± 3.17	subgroup-*p* = 0.059 site-*p* = 0.979 **site × subgroup-*p* = 0.011**
	F_LC	29.03 ± 2.63	25.96 ± 2.25	19.87 ± 2.70	22.42 ± 2.81	
Medial-to-lateral ratio of BV/TV		0.94 ± 0.13	1.32 ± 0.12	1.37 ± 0.11	1.04 ± 0.15	**subgroup-*p*** ** = 0.050**

Analysis of covariance (covariates in the model are evaluated at the following values: age: 66.82 years and BMI: 27.84 kg/m^2^) was used to evaluate potential femoral differences between individuals with different knee alignment (varus and valgus knee in RA and KOA group, subgroup-*p*), investigated skeletal sites (site-*p*), and their interaction (site × subgroup-*p*). Bold font indicates significant differences. For the interpretation of abbreviations used in the table, readers are referred to the list of abbreviations.

**Table 3:** Tibial cartilage and SB microstructural parameters in individuals with RA and KOA depending on knee malalignment.

Skeletal Site		KOA Group Valgus Knee (Mean ± SE)	KOA Group Varus Knee (Mean ± SE)	RA Group Valgus Knee (Mean ± SE)	RA Group Varus Knee (Mean ± SE)	*p*-Value
Cg.Th (mm)	T_MC_A	1.37 ± 0.22	0.63 ± 0.19	1.61 ± 0.20	0.39 ± 0.29	**subgroup-*p* *=* 0.004** site-*p* = 0.059 **site × subgroup-*p* *=* 0.001**
	T_MC_P	2.08 ± 0.24	1.44 ± 0.20	1.31 ± 0.22	0.64 ± 0.26	
	T_LC_A	0.62 ± 0.21	2.25 ± 0.18	0.61 ± 0.19	2.01 ± 0.23	
	T_LC_P	0.80 ± 0.23	2.40 ± 0.19	0.39 ± 0.21	2.00 ± 0.24	
Medial-to-lateral ratio of Cg.Th		2.43 ± 0.53	0.48 ± 0.05	3.12 ± 0.59	0.52 ± 0.08	subgroup-***p* = 0.001**
Ct.Po.tot (%)	T_MC_A	19.47 ± 4.48	14.63 ± 3.84	22.12 ± 4.09	17.34 ± 4.80	subgroup-*p =* 0.676 site-*p =* 0.255 site × subgroup-*p =* 0.150
	T_MC_P	14.91 ± 3.84	14.19 ± 2.31	23.84 ± 2.46	19.80 ± 2.89	
	T_LC_A	15.70 ± 4.20	23.92 ± 3.60	18.44 ± 3.84	20.48 ± 4.50	
	T_LC_P	17.02 ± 3.22	12.56 ± 2.76	20.04 ± 2.94	19.34 ± 3.45	
Ct.Po.Dm (µm)	T_MC_A	110 ± 14	98 ± 12	124 ± 13	136 ± 15	subgroup-*p =* 0.102 site-*p =* 0.651 site × subgroup-*p =* 0.804
	T_MC_P	104 ± 9	101 ± 8	117 ± 9	120 ± 10	
	T_LC_A	103 ± 12	108 ± 10	127 ± 11	111 ± 13	
	T_LC_P	105 ± 13	91 ± 11	133 ± 12	114 ± 14	
Ct.Th (mm)	T_MC_A	0.96 ± 0.16	1.26 ± 0.14	0.64 ± 0.15	1.40 ± 0.18	**subgroup-*p =* 0.030** site-*p =* 0.874 **site × subgroup-*p* *=* 0.001**
	T_MC_P	0.84 ± 0.10	0.99 ± 0.08	0.63 ± 0.09	0.95 ± 0.11	
	T_LC_A	1.40 ± 0.10	0.66 ± 0.09	1.10 ± 0.09	0.63 ± 0.11	
	T_LC_P	1.53 ± 0.20	0.72 ± 0.17	0.89 ± 0.18	0.66 ± 0.21	
Medial-to-lateral ratio of Ct.Th		0.72 ± 0.11	1.25 ± 0.36	0.69 ± 0.04	2.09 ± 0.48	subgroup-***p* = 0.001**
Tb.Th (µm)	T_MC_A	337 ± 196	167 ± 67	193 ± 16	195 ± 21	subgroup-*p* = 0.195 site-*p* = 0.130 site × subgroup-*p* = 0.060
	T_MC_P	217 ± 18	146 ± 15	169 ± 17	169 ± 20	
	T_LC_A	183 ± 17	180 ± 16	179 ± 42	171 ± 19	
	T_LC_P	182 ± 19	182 ± 19	171 ± 17	167 ± 20	
Tb.N (1/mm)	T_MC_A	1.639 ± 0.204	1.625 ± 0.175	1.526 ± 0.186	1.479 ± 0.219	subgroup-*p* = 0.627 site-*p* = 0.422 site × subgroup-*p* = 0.282
	T_MC_P	1.827 ± 0.208	1.634 ± 0.178	1.350 ± 0.190	1.246 ± 0.223	
	T_LC_A	1.471 ± 0.377	1.646 ± 0.324	1.366 ± 0.344	2.561 ± 0.404	
	T_LC_P	1.614 ± 0.177	1.743 ± 0.152	1.421 ± 0.161	1.283 ± 0.189	
BV/TV (%)	T_MC_A	25.34 ± 3.22	18.96 ± 2.57	23.20 ± 2.73	19.78 ± 3.44	**subgroup-*p* = 0.046** site-*p* = 0.975 **site × subgroup-*p* = 0.016**
	T_MC_P	34.49 ± 3.38	19.70 ± 2.99	20.70 ± 3.08	16.88 ± 3.62	
	T_LC_A	22.50 ± 2.99	24.24 ± 2.76	19.40 ± 2.94	22.10 ± 3.20	
	T_LC_P	23.27 ± 2.79	20.65 ± 2.39	20.69 ± 2.54	18.28 ± 2.98	
Medial-to-lateral ratio of BV/TV		1.47 ± 0.13	0.86 ± 0.15	1.10 ± 0.05	0.98 ± 0.12	**subgroup-*p* = 0.004**

Analysis of covariance (covariates in the model are evaluated at the following values: age: 66.82 years and BMI: 27.84 kg/m^2^) was used to evaluate potential tibial differences between individuals with different knee alignments (varus and valgus knee in RA and KOA group, subgroup-*p*), investigated skeletal sites (site-*p*), and their interaction (site × subgroup-*p*). Bold font indicates significant differences. For the interpretation of abbreviations used in the table, readers are referred to the list of abbreviations.

## Data Availability

The original contributions presented in this study are included in the article. Further inquiries can be directed to the corresponding author.

## Abbreviations

The following abbreviations are used in this manuscript:

KOA	Knee osteoarthritis
RA	Rheumatoid arthritis of the knee
SB	Subchondral bone
SBP	Subchondral bone plate
STB	Subchondral trabecular bone
TKA	Total knee arthroplasty
F	Female (women)
M	Male (men)
BMI	Body mass index
HKA	Hip–knee–ankle angle
N.A.	Not assessed (not applicable)
CAD	Coronary artery disease
PVD	Peripheral vascular disease
HLP	Hyperlipidemia
T2DM	Type 2 diabetes mellitus
HT	Hypothyroidism
DMARDs	Disease-modifying anti-rheumatic drugs
F_MC	Medial femoral condyle
F_LC	Lateral femoral condyle
T_MC_A	The anterior part of the medial tibial condyle
T_MC_P	The posterior part of the medial tibial condyle
T_LC_A	The anterior part of the lateral tibial condyle
T_LC_P	The posterior part of the lateral tibial condyle
Ct.Po.tot	Total cortical porosity
Ct.Po.Dm	Cortical pore diameter
Ct.Po.Sp	Cortical pore separation
Ct.Th	Cortical thickness
BV/TV	Bone volume fraction
Tb.Th	Trabecular thickness
Tb.N	Trabecular number
Tb.Sp	Trabecular separation
Conn.Dn	Connectivity density
FD	Fractal dimension
DA	Degree of anisotropy
SMI	Structure model index
Cg.Th	Cartilage thickness
ANCOVA	Analysis of covariance
SD	Standard deviation
SE	Standard error
